# Supplementary material for: Interaction of Cyclosporin C with Dy3+ Ions in Acetonitrile and in Complex with Dodecylphosphocholine Micelles Determined by NMR Spectroscopy
Source: Int J Mol Sci. 2024 Dec 11;25(24):13312. doi: 10.3390/ijms252413312 (PMC11677049; doi:10.3390/ijms252413312)
Supplement: Supplementary file 1 [file ijms-25-13312-s001.zip › ijms-3333030-supplementary.pdf]

Interaction of cyclosporin C with Dy^{3+} ions in acetonitrile and in complex with dodecylphosphocholine micelles determined by NMR spectroscopy (supplementary information)

Artyom S. Tarasov[1], *Guzel A. Minnullina*[1], *Sergey V. Efimov*[1], *Polina P. Kobchikova*[2], *Vladimir V. Klochkov*[1]

[1] *Institute of Physics, Kazan Federal University, 18 Kremlevskaya St., Kazan, 420008 Russian Federation*
[2] *Frank Laboratory of Neutron Physics, Joint Institute for Nuclear Research, 6 Joliot-Curie St., Dubna, Moscow Region, 141980 Russian Federation*

Additional figures related to the study of the system cyclosporin C – DPC micelle in the aqueous medium and to the interaction with other metal ions are gathered in this SI file.

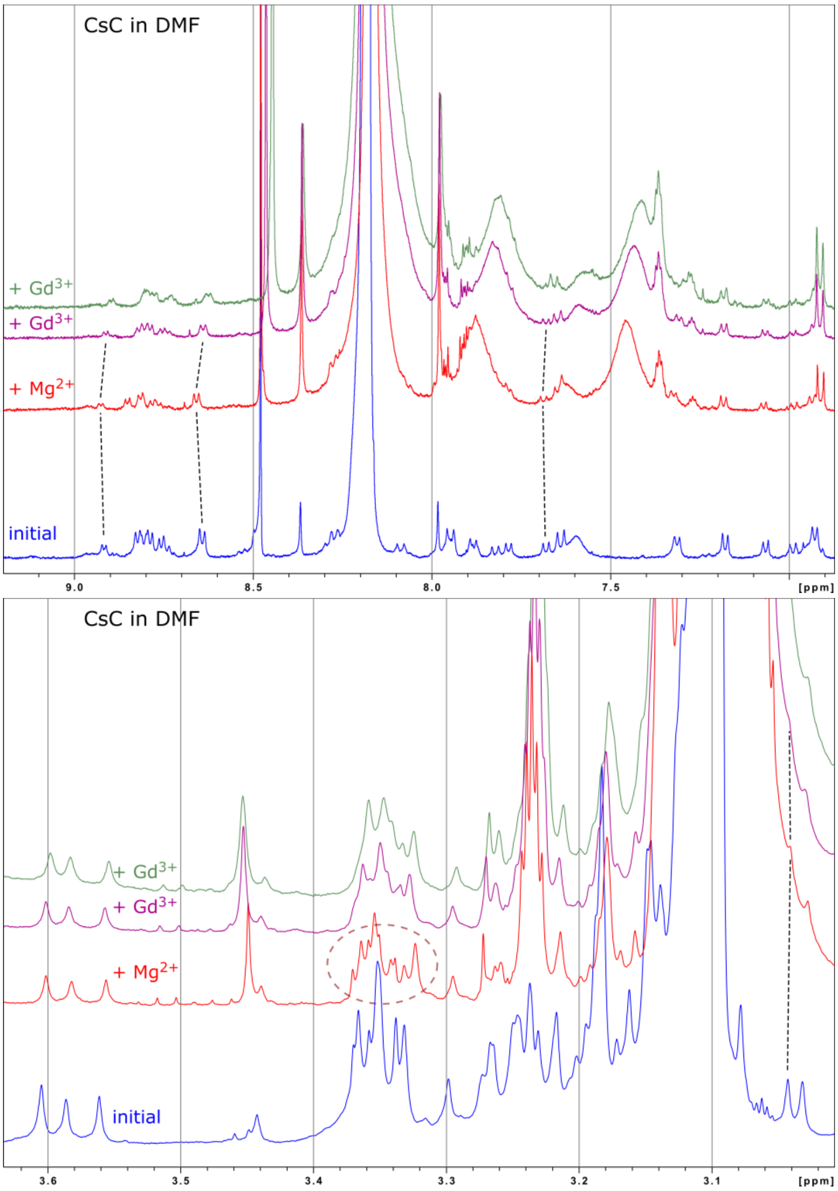

Figure S1. ^{1}H NMR spectrum of CsC in DMF (bottom spectrum, blue) and spectra recorded after addition of an ion: manganese ($MgCl_2$; second spectrum, red) and increasing doses of gadolinium ($Gd(NO_3)_3$; two upper spectra).
Experimental conditions: 500 MHz, 25°C.

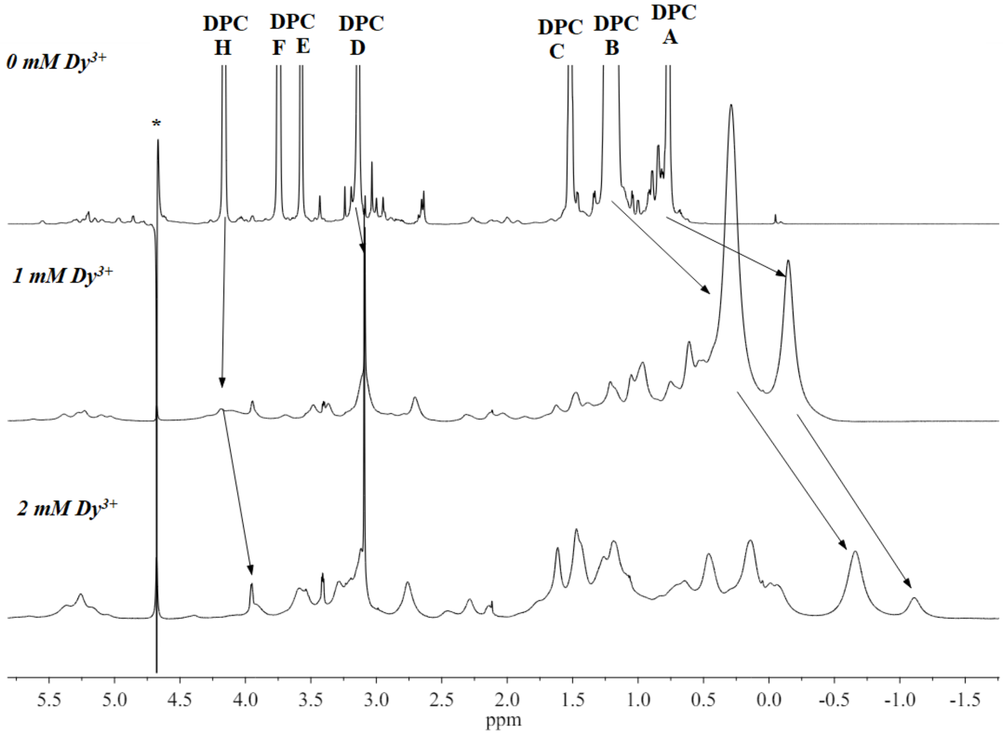

Figure S2. ^{1}H NMR spectrum of CsC – DPC with different Dy(NO$_3$)$_3$ concentrations in D$_2$O. Experimental conditions: 700 MHz, 25°C.

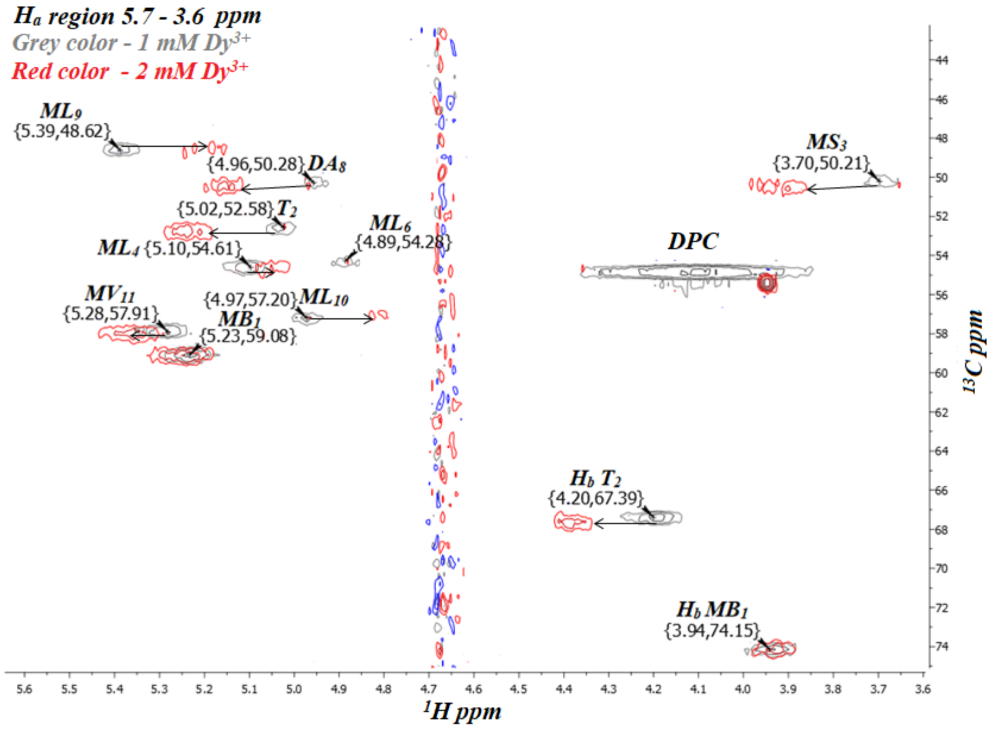

Figure S3. Fragment of ^{1}H-^{13}C HSQC NMR spectrum of CsC – DPC complex with different concentrations of Dy(NO$_3$)$_3$ (red, 1 mM; grey, 2 mM Dy^{3+}) in D$_2$O. Peaks in the spectrum of the sample with 2 mM lanthanide salt broaden and shift further, following the trend started at 0 and 1 mM Dy^{3+} content.

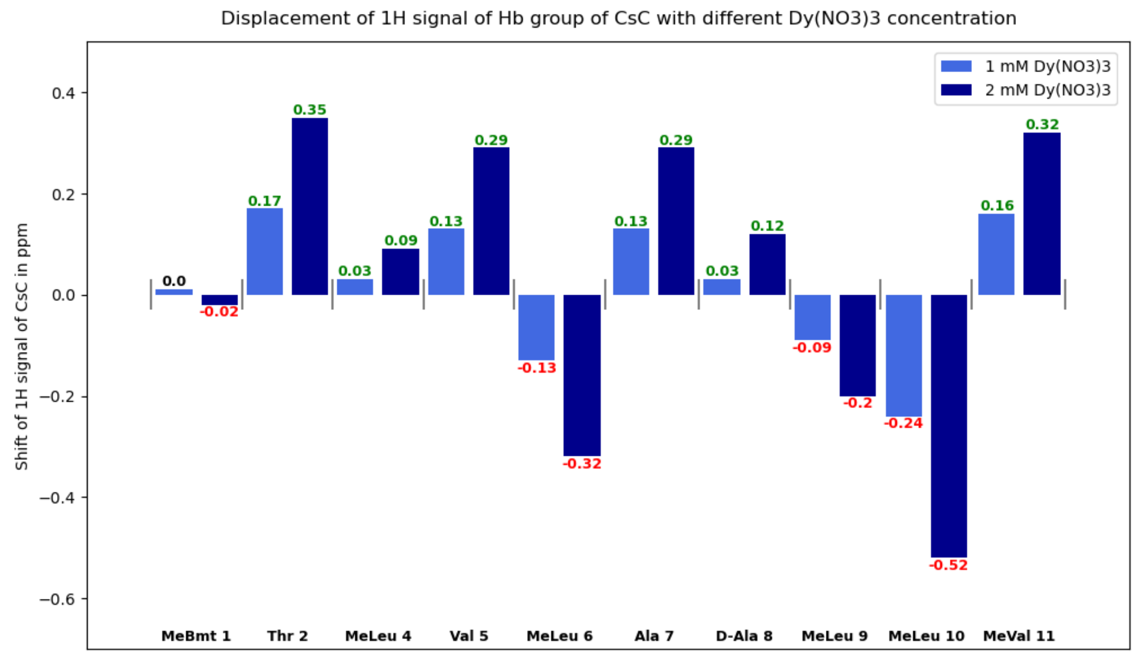

Figure S4. Changes in $\delta(H_\beta)$ of CsC with different $Dy(NO_3)_3$ concentrations.

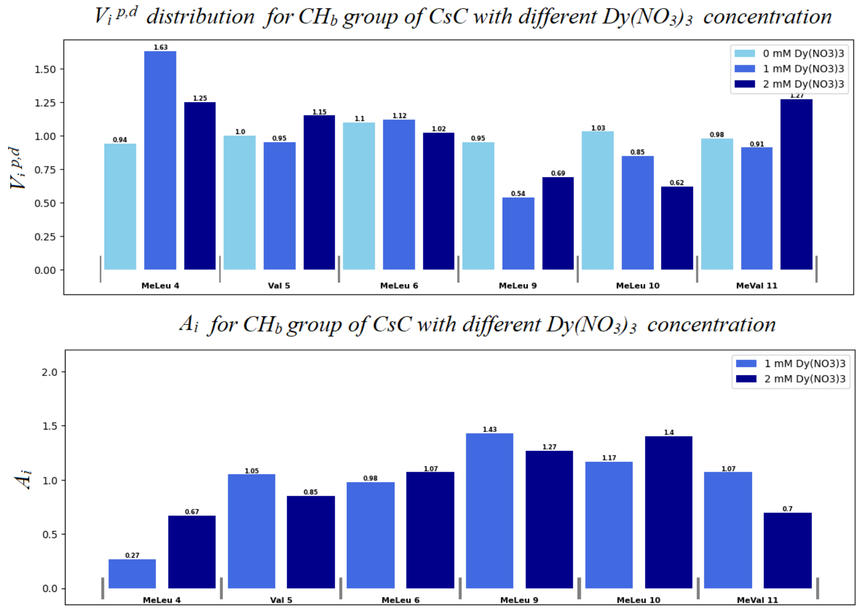

Figure S5. Distribution of autoscaled HSQC volume signals $V_i^{p,d}$ for CHβ protons of CsC. Light blue bars (in the upper panel) correspond to the absence of Dy^{3+} (V_i^{d} values); blue bars, to 1 mM Dy^{3+} (V_i^{p}); dark blue bars, to 2 mM Dy^{3+}.

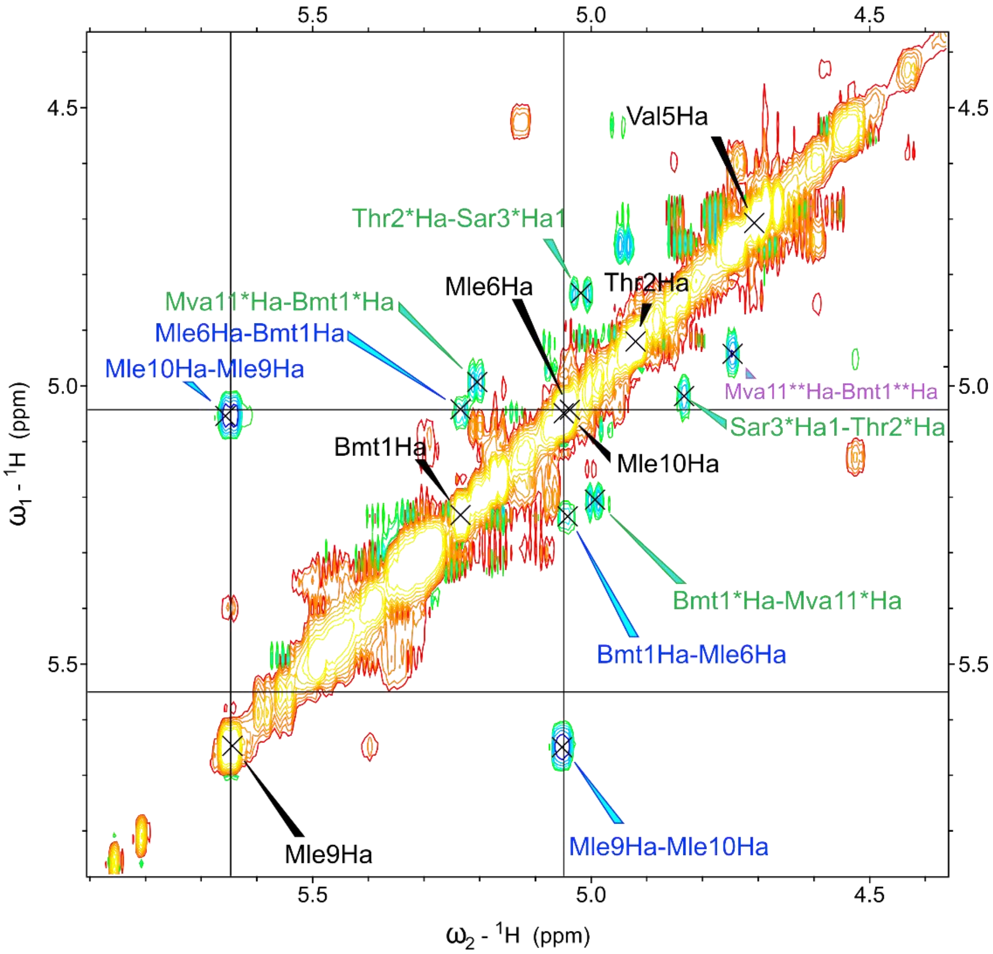

Figure S6. Fragment of ROESY spectrum of CsC in CD_3CN (700 MHz, 25°C, t_{mix} = 250 ms). Shown are cross-peaks between Hα protons indicative of *cis*-bonds between corresponding amino acids (blue labels in the main conformer, green in the second, purple in the third non alalysed here). Red-yellow cross peaks arise due to chemical exchange and are not considered in this work.

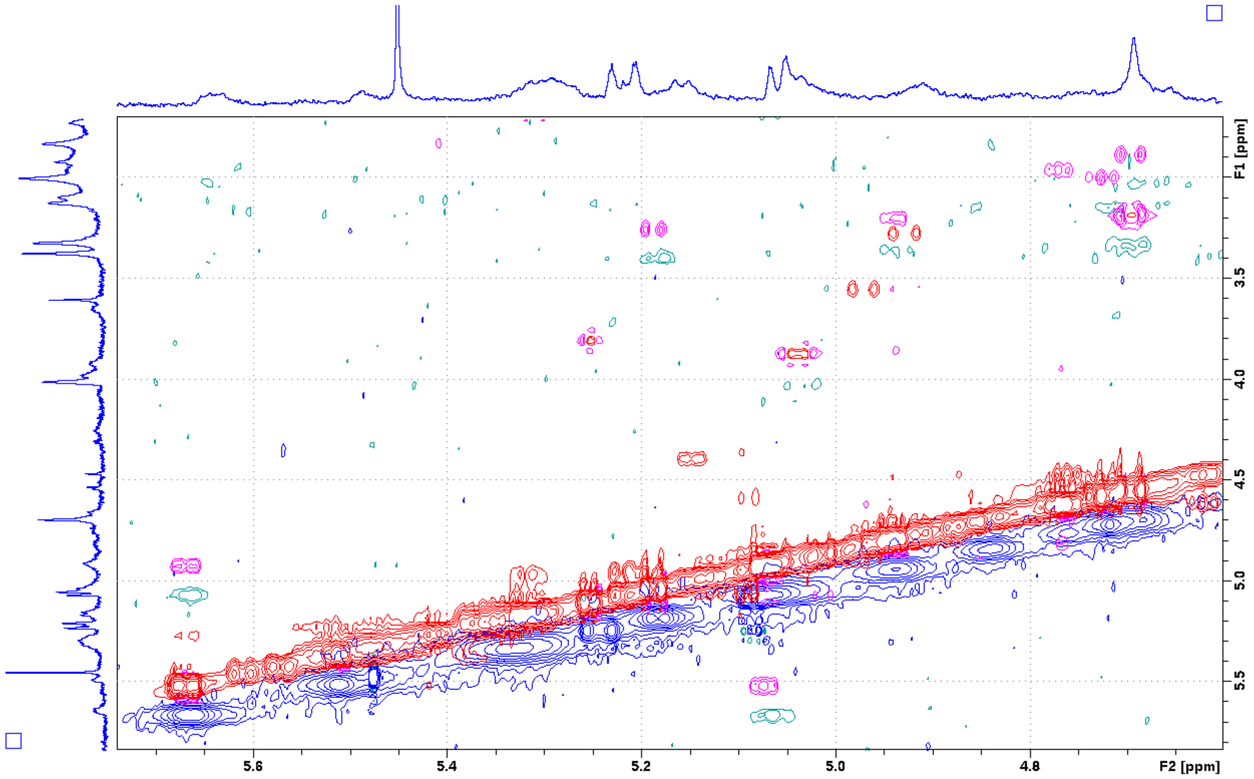

Figure S7. 2D NOESY spectrum of CsC in CD_3CN (700 MHz, t_{mix} = 270 ms, red-pink) and of the same sample with Dy^{3+} added (t_{mix} = 200 ms, blue-green). The first spectrum is shifted along the vertical axis for a better visibility. All NOE peaks (green) have their counterparts in the former spectrum recorded without the metal (pink), but seem more broadened.

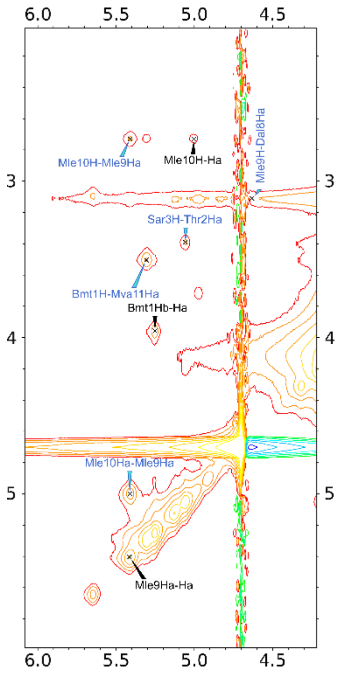

Figure S8. 2D NOESY spectrum of CsC in complex with DPC micelles in water (H_2O + D_2O; 700 MHz, 25°C, t_{mix} = 100 ms, water suppression using noesygpph19 pulse sequence).

Table S1. ^{13}C chemical shifts of CsC with DPC micelles in D_2O at 298 K. Chemical shifts with 1 mM Dy^{3+} added are listed in parentheses ( ); with, 2 mM Dy^{3+}, in square brackets [ ].

**Amino acid residue**	**CN_H / NCH_3 ppm**	**C_α ppm**	**C_β ppm**	**C_γ ppm**	**C_δ ppm**	**Etc ppm**
**$MeBmt_1$**	34.31<br>(34.15)<br>[34.27]	59.28<br>(59.08)<br>[59.17]	74.34<br>(74.15)<br>[74.12]	36.53<br>(36.21)<br>[36.20]	35.70<br>(35.22)<br>[35.14]	**MB_1:**<br>**C_ε:**<br>125.97<br>(-)<br>[-] ,<br>**C_ζ:**<br>130.17<br>(-)<br>[-] ,<br>**C_η:**<br>17.63<br>(-)<br>[-] ,<br>**C_δ':**<br>15.01<br>(14.75)<br>[14.71]
**Thr_2**	-	52.62<br>(52.58)<br>[52.79]	67.46<br>(67.39)<br>[67.73]	18.00<br>(18.01)<br>[18.32]	-	
**$MeSar_3$**	38.37<br>(38.32)<br>[38.60]	50.28<br>(50.28)<br>[50.45]	-	-	-	
**$MeLeu_4$**	30.30<br>(-)<br>[-]	54.73<br>(54.61)<br>[54.69]	35.22<br>(35.26)<br>[35.12]	24.28<br>(24.02)<br>[24.09]	22.10<br>(21.62)<br>[21.46]	
**Val_5**	-	54.62<br>(-)<br>[-]	31.84<br>(31.74)<br>[31.97]	23.39<br>(22.95)<br>[22.60]	-	
**$MeLeu_6$**	30.84<br>(30.38)<br>[30.54]	54.59<br>(54.28)<br>[-]	36.60<br>(36.32)<br>[36.16]	25.23<br>(24.66)<br>[24.72]	22.86<br>(21.84)<br>[-]	
**Ala_7**	-	48.59<br>(-)<br>[-]	16.73<br>(16.67)<br>[16.90]	-	-	
**$D\text{-}Ala_8$**	-	46.16<br>(-)<br>[-]	17.65<br>(17.49)<br>[18.56]	-	-	
**$MeLeu_9$**	29.28<br>(29.26)<br>[29.50]	48.88<br>(48.62)<br>[48.61]	38.73<br>(38.35)<br>[38.29]	24.34<br>(23.98)<br>[24.02]	23.41<br>(23.39)<br>[22.60]	
**$MeLeu_{10}$**	30.00<br>(29.80)<br>[29.89]	57.51<br>(57.20)<br>[56.95]	41.48<br>(41.01)<br>[40.75]	24.99<br>(24.81)<br>[24.41]	20.61<br>(20.50)<br>[20.58]	
**$MeVal_{11}$**	2.65<br>(2.71)<br>[2.76]	5.20<br>(5.28)<br>[5.36]	2.12<br>(2.28)<br>[2.44]	0.84; 0.79<br>(1.05; 0.96)<br>[1.26;1.16]	-	
